# Clinically Diagnosed Decompensated Chronic Liver Disease With Suspected Portal Hypertension in a One‐Year‐Old Infant: Diagnostic Challenges in a Resource‐Limited Setting

**DOI:** 10.1002/ccr3.73343

**Published:** 2026-08-19

**Authors:** Khadar Jama Ibrahim, Abdisamed Mohamoud H. Ali, Nouradin Ibrahim Omer, Omer Ali Elmi

**Affiliations:** ^1^ College of Health Sciences, School of Medicine and Surgery Amoud University Borama Somaliland; ^2^ Emergency Department Hargeisa Group Hospital Hargeisa Somaliland; ^3^ College of Medicine and Surgery, University of Hargeisa Hargeisa Somaliland; ^4^ Department of Pediatrics Hargeisa Group Hospital Hargeisa Somaliland

**Keywords:** case report, chronic liver disease, differential diagnosis, infant, portal hypertension, resource‐limited setting

## Abstract

Chronic liver disease in infancy is uncommon, diagnostically demanding, and rarely reported from sub‐Saharan Africa, where advanced hepatologic evaluation is seldom accessible. We describe a one‐year‐old boy from rural Somaliland who presented with a two‐week history of progressive abdominal distension, followed by jaundice, hematemesis and bloody diarrhea. Examination showed wasting, deep scleral icterus, tense ascites, caput medusae and splenomegaly. Investigations demonstrated severe anemia (hemoglobin 3.8 g/dL), thrombocytopenia (130 × 10^9^/L), conjugated hyperbilirubinemia (total 2.45 mg/dL, direct 1.69 mg/dL), severe hypoalbuminemia (albumin 1.8 g/dL) and marked coagulopathy (INR 3.0). Grayscale ultrasonography showed an irregular hepatic surface, splenomegaly and large‐volume ascites. Doppler ultrasonography, upper gastrointestinal endoscopy, liver biopsy, elastography, cytomegalovirus IgM and polymerase chain reaction testing, and metabolic and genetic studies were unavailable, and an isolated positive cytomegalovirus IgG was considered inconclusive. A clinical diagnosis of decompensated chronic liver disease with suspected portal hypertension was therefore made without an established etiology. Whole blood transfusion, intravenous albumin, vitamin K, sodium restriction and combined spironolactone and furosemide produced short‐term improvement, with a 4 cm reduction in abdominal girth, a rise in hemoglobin to 8.3 g/dL and cessation of bleeding. This case shows that a defensible syndromic diagnosis and effective stabilization are achievable without advanced testing, and supports adoption of a defined minimum diagnostic dataset—fractionated bilirubin, gamma‐glutamyl transferase, coagulation profile, albumin, hepatitis serology, diagnostic paracentesis and grayscale ultrasonography—for infants with suspected chronic liver disease in low‐resource settings.

## Key Clinical Message

In infants with ascites, splenomegaly and gastrointestinal bleeding, portal hypertension can be diagnosed clinically when Doppler, endoscopy and biopsy are unavailable. Pragmatic supportive care may stabilize the child, but the underlying cause remains unproven and should be reported as suspected rather than confirmed.

## 1 Introduction

Chronic liver disease (CLD) in children represents a heterogeneous group of disorders that may progress to cirrhosis and life‐threatening complications. Although relatively uncommon in infancy, CLD carries significant morbidity and mortality, particularly when diagnosis is delayed until decompensation occurs [1, 2]. Decompensated cirrhosis is defined by complications such as ascites, jaundice, variceal bleeding and impaired hepatic synthetic function, all of which indicate advanced and often irreversible liver damage [3].

Globally, the causes of pediatric CLD vary by region. In high‐income countries genetic and metabolic disorders predominate, whereas in low‐resource settings infectious diseases, malnutrition and incomplete immunization remain major contributors [4]. Portal hypertension in children differs from the adult disease in both etiology and management. Extrahepatic portal vein obstruction predominates in low‐ and middle‐income countries, whereas biliary atresia and other cholestatic disorders account for most intrahepatic disease presenting in the first year of life [5, 6, 7]. Distinguishing between the two carries major prognostic consequences, yet the investigations required to do so are precisely those least available in resource‐constrained hospitals [8, 9].

Published data from sub‐Saharan Africa describing the presentation and outcome of infants with advanced liver disease remain limited. This report describes a clinically diagnosed decompensated chronic liver disease with suspected portal hypertension in a one‐year‐old infant in Somaliland, setting out the clinical reasoning, the differential diagnoses that could not be excluded, and the diagnostic ceiling imposed by the setting.

## 2 Case Presentation

A 1‐year‐old male infant from rural Somaliland presented with a two‐week history of progressive abdominal distension. The distension increased gradually and became associated with visibly prominent abdominal wall veins. During this period the child experienced intermittent episodes of fever.

Two days prior to presentation the child developed sudden‐onset vomiting, occurring approximately three times per day. The vomiting was of moderate volume and was intermittently mixed with small amounts of blood. This was accompanied by bloody diarrhea, poor feeding and loss of appetite. The caregiver also noted recent onset of yellow discoloration of the eyes. A mild cough was reported, without associated difficulty in breathing.

There was no history of convulsions, pruritus, dark urine, pale stools, easy bruising or prolonged bleeding. There were no features suggestive of hepatic encephalopathy, such as irritability, lethargy, excessive sleepiness or altered responsiveness. Stool colour was reported by the caregiver rather than observed by the clinical team, and this history is therefore of limited reliability.

The child resides in a rural setting characterized by poor sanitation and limited access to healthcare. There is a history of recurrent gastrointestinal illnesses, and the child had not received any routine immunizations, including hepatitis B vaccination. There was no reported history of medication use, herbal remedies, toxin exposure or ingestion of contaminated food prior to the onset of illness.

At 4 months of age the child had an episode of jaundice that resolved spontaneously without medical evaluation. Since infancy the child had been admitted three times for episodes of vomiting and diarrhea. The child was born via normal spontaneous vaginal delivery with no perinatal complications. According to the mother, the child had intermittent umbilical discharge during the neonatal period, which was not medically evaluated or treated. There was no documented history of neonatal hepatitis. The child was initially breastfed but underwent early and inconsistent weaning, with limited dietary diversity. In the days preceding admission the caregiver reported reduced oral intake and poor feeding tolerance.

Developmental milestones were age‐appropriate, and there was no family history of liver, metabolic or bleeding disorders. On review of systems, the mother reported decreased activity and weight loss before presentation; other systems were unremarkable.

### 2.1 Physical Examination

On examination the infant appeared chronically ill, hypoactive and significantly wasted. Vital signs on admission were temperature 36.5°C, heart rate 116 beats/min, respiratory rate 38 breaths/min and oxygen saturation 98% on room air. Anthropometric measurements at admission were weight 7.2 kg, length 70 cm, mid‐upper arm circumference 11.2 cm and head circumference 45 cm. The recorded weight is likely to underestimate the true degree of wasting because of the ascitic fluid burden.

**FIGURE 1:**
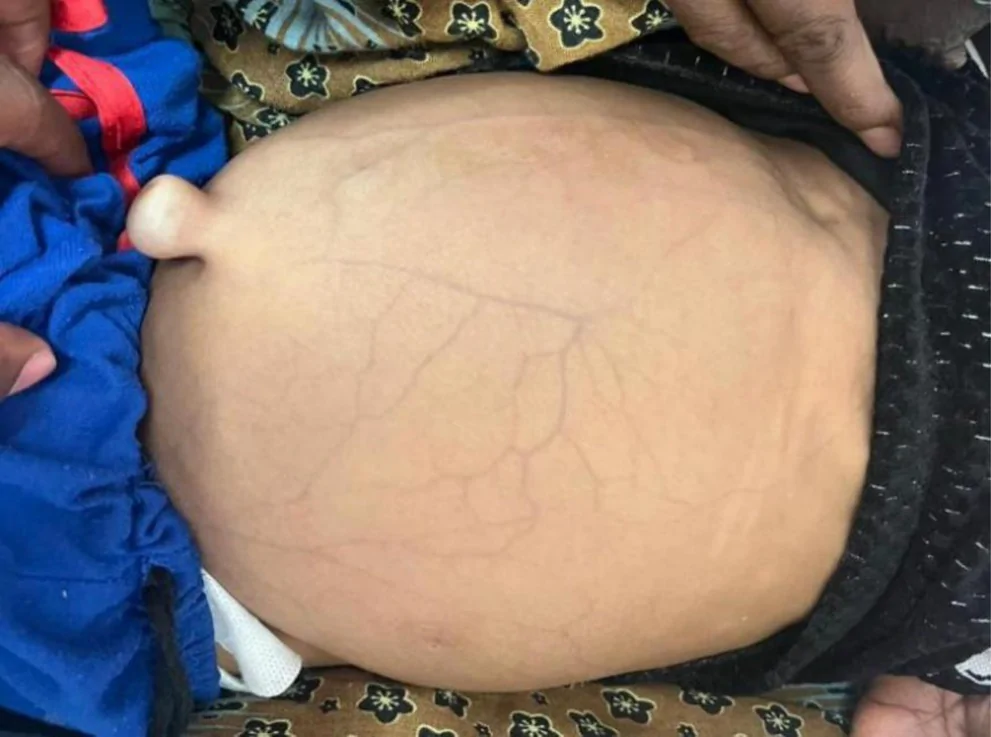
Clinical photograph showing a globally distended abdomen with bulging flanks and visible tortuous veins (caput medusae) on the anterior abdominal wall. Abdominal girth at the time of photography was 54 cm, which provides the scale reference; no calibrated scale bar was in the field of view at acquisition. The image was cropped only to remove identifying features.

Examination of the head and neck demonstrated deep scleral icterus and oral thrush. Abdominal examination revealed marked global distension with an abdominal girth of 54 cm, prominent tortuous peri‐umbilical veins consistent with caput medusae, and a positive fluid wave with shifting dullness suggestive of large‐volume ascites. Splenomegaly was palpable, while the liver was difficult to assess because of marked abdominal tenseness. No peripheral edema or petechiae were noted; however, the child had striking pallor consistent with severe anemia. The clinical findings are illustrated in Figure 1.

### 2.2 Laboratory Findings

The laboratory investigations are summarized in Table 1. Serum gamma‐glutamyl transferase, alkaline phosphatase and serum bile acids were not performed, and diagnostic paracentesis was not undertaken.

**TABLE 1:** Laboratory findings at admission with interpretation.

Test	Result	Reference range	Interpretation
Hemoglobin	3.8 g/dL	10.5–13.5 g/dL	Severe normocytic anemia
White blood cell count	13 × 10^9^/L	5.0–12.0 × 10^9^/L	Mild leukocytosis
Platelets	130 × 10^9^/L	150–450 × 10^9^/L	Mild thrombocytopenia; compatible with hypersplenism
Total bilirubin	2.45 mg/dL (41.9 μmol/L)	< 1.0 mg/dL	Hyperbilirubinemia
Direct bilirubin	1.69 mg/dL (28.9 μmol/L)	< 0.3 mg/dL	Conjugated hyperbilirubinemia, indicating hepatobiliary rather than hemolytic jaundice
Albumin	1.8 g/dL (18 g/L)	3.5–5.4 g/dL	Severe hypoalbuminemia; impaired synthetic function, with malnutrition contributory
AST	72 U/L	15–60 U/L	Mildly elevated; modest relative to the degree of synthetic failure
ALT	57 U/L	5–60 U/L	Mildly elevated
Creatinine	0.22 mg/dL	0.2–0.4 mg/dL	Normal; no renal dysfunction
ESR	30 mm/h	< 10–20 mm/h	Elevated; non‐specific
HAV IgM, HBsAg, HCV Ab	All negative	Negative	Hepatitis A, B and C not supported as the primary etiology
CMV IgG	Positive	Negative	Indicates prior exposure only. Without IgM or PCR this result is inconclusive and cannot establish causation
Prothrombin time	Prolonged	Age‐appropriate range	Impaired synthetic function and/or vitamin K deficiency
INR	3.0	0.8–1.2	Marked coagulopathy

### 2.3 Imaging

**FIGURE 2:**
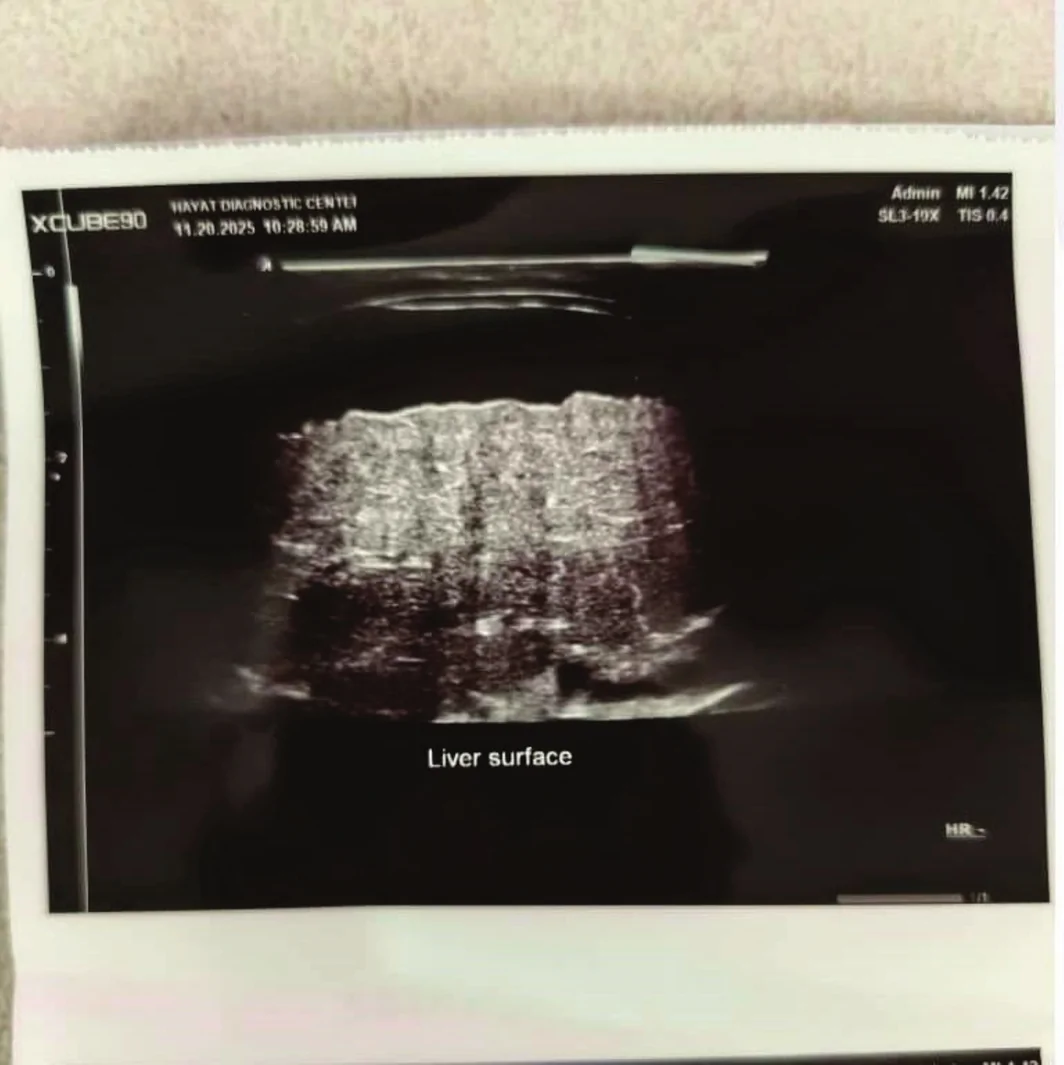
Grayscale abdominal ultrasonography demonstrating an irregular hepatic surface, consistent with chronic parenchymal liver disease, adjacent to anechoic free intraperitoneal fluid. The calibrated depth scale generated by the ultrasound system is displayed along the image margin and serves as the scale reference.

Abdominal ultrasonography demonstrated an irregular hepatic surface, a finding suggestive of chronic parenchymal liver disease although neither sensitive nor specific for cirrhosis in infancy [4, 6]. The gallbladder was mildly distended with diffuse wall thickening, a recognized non‐specific consequence of hypoalbuminemia and ascites rather than an indicator of primary biliary pathology. A large volume of free peritoneal fluid was present, consistent with ascites, and splenomegaly was confirmed (Figure 2). Grayscale ultrasonography did not demonstrate frank portal vein thrombosis; however, dedicated Doppler assessment of portal venous flow and patency was not performed, so a thrombotic or extrahepatic portal venous process cannot be excluded.

## 3 Diagnostic Assessment and Differential Diagnosis

The combination of tense ascites, caput medusae, splenomegaly, thrombocytopenia and upper gastrointestinal bleeding constitutes a clinical syndrome of portal hypertension. Where hepatic venous pressure gradient measurement and endoscopy are unavailable, such clinical and sonographic surrogates are accepted as the practical basis for diagnosis while remaining inferential [4, 6].

The central question was whether the portal hypertension was intrahepatic or extrahepatic, since the two carry different prognoses. Extrahepatic portal vein obstruction is a common cause of pediatric portal hypertension in low‐ and middle‐income countries and is a recognized sequela of neonatal umbilical sepsis, which this child's history raised as a plausible mechanism [7, 10]. However, extrahepatic obstruction characteristically spares hepatic synthetic function; although subclinical dysfunction has been described in long‐standing disease, an albumin of 1.8 g/dL with an INR of 3.0 exceeds what isolated extrahepatic obstruction would be expected to produce [9, 11]. Together with conjugated hyperbilirubinemia and an irregular hepatic surface, these findings pointed to intrahepatic parenchymal disease as the dominant process. The working diagnosis rests on this reasoning rather than on any confirmatory test. The differential diagnoses considered are set out in Table 2.

**TABLE 2:** Differential diagnosis of infantile chronic liver disease with portal hypertension in this patient.

Differential diagnosis	Reasoning in this patient	Confirmatory test (unavailable)
Late‐presenting biliary atresia or other neonatal cholestatic disorder	Supported by conjugated hyperbilirubinemia and jaundice at 4 months; against by the absence of reported acholic stools and by spontaneous resolution of that episode	Liver biopsy, intraoperative cholangiography
Extrahepatic portal vein obstruction after neonatal omphalitis	Supported by the history of umbilical discharge, splenomegaly and hematemesis; against by hypoalbuminemia and INR 3.0, which exceed what isolated extrahepatic obstruction typically produces	Doppler ultrasonography, CT or MR portography
Congenital hepatic fibrosis	Supported by portal hypertension with only modest transaminase elevation; against by the severity of synthetic failure	Liver biopsy
Inherited metabolic liver disease (tyrosinemia Type 1, galactosemia, glycogen storage disease Type IV, alpha‐1 antitrypsin deficiency)	Supported by infantile onset, failure to thrive, and coagulopathy disproportionate to transaminase elevation; against by age‐appropriate development and absence of family history	Plasma amino acids, urine succinylacetone, alpha‐1 antitrypsin phenotype, genetic panel
Congenital or perinatally acquired CMV hepatitis	Raised by positive CMV IgG in an endemic setting; IgG alone indicates exposure and cannot date the infection or establish causation	CMV IgM, IgG avidity, blood or urine PCR, liver histology
Chronic hepatitis B or C	Raised by absent immunization and high regional prevalence; HBsAg and HCV antibody negative, although antibody testing may be unreliable in infancy	HBV DNA, HCV RNA
Hepatosplenic schistosomiasis	Regionally relevant, but the patient is below the usual latency period for fibrosis and had no documented freshwater exposure	Stool or urine microscopy, serology
Non‐hepatic causes of ascites (tuberculous peritonitis, nephrotic syndrome, cardiac failure)	Argued against by conjugated hyperbilirubinemia, coagulopathy, splenomegaly and an irregular hepatic surface, all of which localize the process to the liver	Diagnostic paracentesis with serum–ascites albumin gradient, urinalysis, echocardiography

Abbreviation: CMV, cytomegalovirus.

The diagnosis reached was decompensated chronic liver disease of undetermined etiology with clinically diagnosed portal hypertension. This is a syndromic diagnosis supported by consistent clinical, biochemical and sonographic findings; it is not an etiologic diagnosis, and no entry in Table 2 was confirmed.

## 4 Therapeutic Intervention

Following admission the child was stabilized and resuscitated with intravenous fluids according to standard pediatric protocols. Empirical broad‐spectrum intravenous antibiotics were initiated. Vitamin K was administered to address the coagulopathy, and intravenous albumin was given for hypoalbuminemia and ascites. Given the significant abdominal distension and suspected portal hypertension, diuretic therapy was commenced with spironolactone 2 mg/kg/day and furosemide 1 mg/kg/dose every 12 h. One unit of whole blood was transfused at 10 mL/kg for anemia. Dietary sodium restriction was implemented to assist ascites control, and a nutritional assessment was performed with anthropometric measurements documented at admission as detailed above.

## 5 Follow‐Up and Outcomes

**FIGURE 3:**
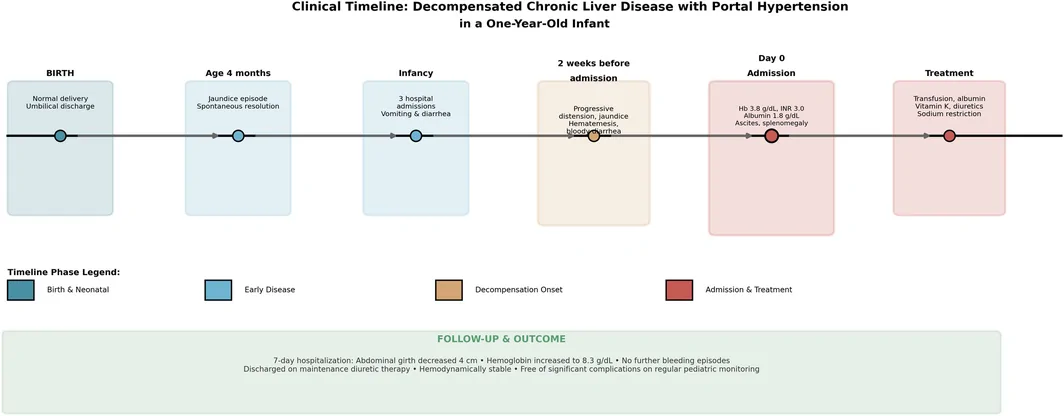
Timeline of the clinical course, from birth through hospital admission, treatment and outpatient follow‐up. This figure is a schematic diagram; a scale bar is not applicable, and intervals are labeled explicitly rather than drawn to a linear time scale.

The patient completed a 7‐day hospitalization with a favorable clinical course. After 1 week of combined diuretic therapy and intravenous albumin, abdominal girth decreased by 4 cm, jaundice partially regressed and oral intake improved. Hemoglobin rose to 8.3 g/dL following whole blood transfusion. No further episodes of hematemesis or bloody diarrhea occurred, and the patient remained hemodynamically stable and afebrile throughout admission. No adverse treatment‐related events were recorded. The patient was discharged on maintenance diuretic therapy with spironolactone and furosemide, and at the most recent outpatient review remained free of significant complications under regular pediatric monitoring. The clinical course is summarized in Figure 3.

## 6 Discussion

Decompensated chronic liver disease in infancy is rare and carries substantial morbidity and mortality, particularly when presentation is delayed until complications such as ascites, jaundice, gastrointestinal bleeding and impaired synthetic function become clinically evident [3]. This patient presented with tense ascites, caput medusae, splenomegaly, thrombocytopenia, severe hypoalbuminemia and coagulopathy, a constellation characteristic of, but not diagnostic of, portal hypertension with advanced chronic liver dysfunction [2, 12, 13]. Where invasive portal pressure assessment is unavailable, clinical and ultrasonographic findings remain the principal surrogate markers, and their limitations must be acknowledged when they form the sole basis for diagnosis [6, 12].

The positive CMV IgG requires cautious interpretation and does not support a causal inference. IgG indicates prior exposure at an undetermined time and distinguishes neither congenital from acquired nor active from remote infection [14, 15]. Passively acquired maternal antibody is a less likely explanation at 12 months of age but cannot be formally excluded without paired maternal testing, and in a population with high seroprevalence the finding carries limited discriminatory value [16]. Confirmation of congenital infection requires detection of viral DNA in early infancy, which was not possible here. CMV IgM, IgG avidity and polymerase chain reaction were unavailable, and no liver tissue was obtained. The result is therefore inconclusive, and cytomegalovirus is reported as one unconfirmed candidate etiology rather than a probable contributing cause [14].

The reported episode of jaundice at 4 months raises concern for an untreated neonatal cholestatic disorder, including late‐presenting biliary atresia or idiopathic neonatal hepatitis; conjugated hyperbilirubinemia persisting beyond 2 weeks of age is always pathological and mandates urgent evaluation, an opportunity missed in this child [5, 17]. The report of neonatal umbilical discharge introduces the possibility of omphalitis complicated by pylephlebitis and portal vein thrombosis, a recognized cause of extrahepatic portal hypertension in infancy, although the severity of synthetic impairment argues against this being the sole process [7, 11]. Although initial viral hepatitis serology was negative, vertically acquired hepatitis C cannot be definitively excluded, as antibody testing may be unreliable in the first year of life. Schistosomiasis was considered because of regional relevance but judged unlikely given the patient's young age, the absence of documented freshwater exposure, and the preservation of synthetic function that typically characterizes periportal fibrosis.

The consequences of this uncertainty are clinically significant. Tyrosinemia Type 1 is specifically treatable and characteristically produces coagulopathy disproportionate to transaminase elevation, the pattern seen here. Extrahepatic portal vein obstruction carries a substantially better hepatic prognosis and would direct management toward variceal surveillance rather than transplant assessment [9, 10]. Neither could be pursued, and the inability to distinguish a potentially treatable disorder from irreversible parenchymal disease is the central difficulty this case illustrates.

Management was supportive and aligned with established recommendations for pediatric decompensated liver disease [18]. Diuretic therapy with spironolactone and furosemide remains central to ascites control, while albumin infusion is beneficial in severe hypoalbuminemia and circulatory dysfunction [2]. The reduction in abdominal girth and clinical stabilization following transfusion, albumin and diuretic therapy suggest short‐term effectiveness of pragmatic supportive management despite limited resources. This inference should be treated cautiously: the improvement was observed over a single seven‐day admission in one patient, and it demonstrates short‐term symptomatic response rather than any effect on the natural history of the disease.

This case highlights the realities of pediatric hepatology practice in low‐resource settings, where financial constraints and the absence of advanced investigations often restrict care to stabilization rather than etiologic diagnosis. Several of the investigations not performed require no advanced infrastructure: diagnostic paracentesis with a serum–ascites albumin gradient, serum gamma‐glutamyl transferase and stool occult blood testing are inexpensive and were achievable in this hospital. Their addition to a minimum dataset for infants with suspected chronic liver disease would narrow the differential diagnosis at negligible cost [5]. Given the guarded prognosis in the absence of definitive treatment such as liver transplantation, strengthening early detection, hepatitis B immunization, nutritional support and referral pathways for infantile cholestatic disease remains essential [4].

## 7 Limitations

The principal limitation is that the diagnosis is clinical and syndromic rather than confirmed. Doppler ultrasonography was unavailable, so portal vein patency and flow direction were never assessed and intrahepatic disease could not be formally separated from extrahepatic portal vein obstruction—the distinction that would most have altered prognosis and management. Upper gastrointestinal endoscopy was unavailable, so varices were never visualized and the source of bleeding is inferred rather than established. Liver biopsy was unavailable, so cirrhosis was neither confirmed nor distinguished from congenital hepatic fibrosis or a storage disorder; this is the principal reason the diagnosis is described as clinical rather than confirmed. CMV IgM, avidity testing and polymerase chain reaction were unavailable, so exposure could not be dated or linked causally to the liver disease. Metabolic and genetic testing were unavailable, leaving tyrosinemia Type 1—a treatable disorder consistent with this biochemical pattern—formally unexcluded, which is the most clinically significant gap in the evaluation.

Two omissions warrant acknowledgment because they were feasible within our institution: diagnostic paracentesis, which would have confirmed portal hypertensive ascites and excluded spontaneous bacterial and tuberculous peritonitis, and serum gamma‐glutamyl transferase, which would have helped separate high‐GGT from low‐GGT cholestasis at minimal cost. Several elements of the history—neonatal umbilical discharge, the episode of jaundice at 4 months and stool color—rest on caregiver report and could not be independently verified. Follow‐up is short, precluding assessment of disease trajectory or survival, and as a single case report these observations support no generalizable inference regarding the epidemiology, management or outcome of infantile chronic liver disease in this region.

## 8 Conclusion

This case illustrates the diagnostic and therapeutic challenges of clinically diagnosed decompensated chronic liver disease with suspected portal hypertension in infancy in a resource‐limited setting. A defensible syndromic diagnosis was reached and effective short‐term stabilization achieved using clinical assessment, basic laboratory testing and grayscale ultrasonography alone; the etiology was never established, and treatable causes could not be excluded. Clinicians working without advanced diagnostics should report such cases as suspected or clinically diagnosed and state explicitly which alternatives remain open. A small and inexpensive set of investigations—fractionated bilirubin, gamma‐glutamyl transferase, coagulation profile, albumin, hepatitis serology and diagnostic paracentesis—would substantially narrow the differential diagnosis in comparable patients and should be established as the minimum standard, alongside continued advocacy for pediatric hepatology capacity, immunization coverage and referral pathways.

## 9 Patient Perspective

The infant's primary caregiver (mother) was interviewed regarding her experience during the child's illness and hospitalization. She reported significant psychological distress related to the child's progressive clinical deterioration and to the financial difficulties associated with seeking medical care. Following the child's clinical improvement and hospital discharge, she expressed satisfaction with the supportive management provided. However, she also expressed concern regarding the inability to complete recommended diagnostic investigations, including metabolic, genetic and endoscopic evaluations, because of financial constraints.

## Author Contributions

**Omer Ali Elmi:** project administration, supervision, resources, validation. **Khadar Jama Ibrahim:** conceptualization, investigation, methodology, writing – original draft. **Abdisamed Mohamoud H. Ali:** funding acquisition, visualization, validation. **Nouradin Ibrahim Omer:** methodology, formal analysis, data curation.

## Funding

The authors have nothing to report.

## Consent

Written informed consent was obtained from the patient's legal guardian for publication of this case report and any accompanying images. A copy of the written consent is available for review by the Editor‐in‐Chief of this journal.

## Conflicts of Interest

The authors declare no conflicts of interest.

## Data Availability

The data supporting the findings of this case report are contained within the article. Additional information is available from the corresponding author upon reasonable request, while maintaining patient confidentiality.
